# Tactile breathing guidance increases oxygen saturation but not alertness or hypoxia symptoms

**DOI:** 10.1371/journal.pone.0302564

**Published:** 2024-06-12

**Authors:** Yuval Steinman, Eric Groen, Monique H. W. Frings-Dresen

**Affiliations:** 1 Center for Man in Aviation, Royal Netherlands Air Force, Soesterberg, The Netherlands; 2 Department Public and Occupational Health/Coronel Institute of Occupational Health, Amsterdam University Medical Centers Location University of Amsterdam, Amsterdam, The Netherlands; 3 Department of Human Performance, TNO, Soesterberg, The Netherlands; 4 Safety and Accident Investigation Centre, Cranfield University, Cranfield, United Kingdom; Bangor University, UNITED KINGDOM

## Abstract

We investigated the effect of tactile guided slow deep breathing compared with that of spontaneous breathing on blood oxygen saturation (SpO_2_), alertness, and hypoxia symptoms during acute hypobaric hypoxia. We also evaluated the usability of this tactile breathing guidance. Twelve male military pilots were exposed to a simulated altitude of 4,572 m (15,000 ft) in a repeated measures study while breathing spontaneously and during tactile guided slow deep breathing. Under both breathing conditions, measurements were performed at rest and during the performance of a cognitive task. The Stanford Sleepiness Scale was used to rate alertness, and hypoxia symptoms were reported using a list of general hypoxia symptoms. Usability was evaluated in a questionnaire. Tactile guidance of slow deep breathing significantly increased (*p* <.001) the SpO_2_ – 88% (95% confidence interval (CI) [84%, 91%]) at rest and 85% (95% CI [81%, 88%]) during the cognitive task – compared with spontaneous breathing – 78% (95% CI [75%, 81%]) at rest and 78% (95% CI [76%, 80%]) during the cognitive task. This increase in SpO_2_ had no effect on the level of alertness and number of hypoxia symptoms. Pilots were positive about the intensity and sensation of the vibration signal, but had difficulty following the vibration pattern during the cognitive task. Pre-training may improve slow deep breathing technique during performance of cognitive tasks.

## Introduction

During flight at altitude, one threat to the flight performance of helicopter crewmembers is hypobaric hypoxia [1, 2]. Hypobaric hypoxia is a state of insufficient oxygen in the body tissue that is caused by a decrease in atmospheric pressure. This decrease reduces the oxygen tension in inspired air, which in turn reduces the oxygen tension in the arterial blood [3]. The body’s initial reaction to a decrease in arterial oxygen tension is to increase cardiac output and respiratory rate, both of which aim to increase arterial blood oxygenation and support cerebral oxygen delivery [4]. However, the increased respiratory rate has been shown to reduce the partial pressure of carbon dioxide in arterial blood [5]. This can decrease cerebral oxygen levels by inciting cerebral vasoconstriction and decreasing oxygen unloading [6, 7].

Previous studies have shown that slow deep breathing (3–6 breaths/min) during hypoxia significantly increases arterial oxygen saturation compared with spontaneous breathing and this type of breathing has been suggested as an effective technique to cope with the effects of altitude-induced hypoxia [8, 9]. The larger tidal volume (V_T_) during slow deep breathing improves ventilation efficiency by reducing anatomical dead space in the lungs, optimizing alveolar ventilation, and improving blood flow to lung areas that are normally less well perfused [10].

Military helicopter crewmembers fly at altitude in unpressurized aircraft that are not always equipped with an oxygen system. They are trained to recognize hypoxia symptoms and descend immediately to a flight level where their arterial oxygen saturation can increase sufficiently to eliminate these symptoms. Slow deep breathing can help helicopter crewmembers increase their arterial oxygen saturation. However, a recent study [11] has shown that helicopter crewmembers engaged in a (military) flight task ignored their hypoxia symptoms and were not aware of becoming hypoxic. This indicates that crewmembers may not start slow deep breathing in time, and that an external warning signal could be useful to prompt crewmembers to initiate slow deep breathing and to help them maintain an optimal breathing rhythm.

In-flight warnings are usually relayed to the crewmembers visually (via cockpit instruments) or aurally (via voice warnings). However, hypoxia can affect vision [12–14] and hearing [15]. In contrast, neural activities of somatosensory processing (processing of touch receptor signals in the body by the brain) do not seem to be sensitive to hypoxia [16], so tactile signals (based on touch) may be better for relaying essential information. During driving, for example, tactile feedback has been shown to be more effective than auditory feedback at relaying warnings [17]. In addition, an experiment performed by the US Navy [18] has shown that tactile feedback can help pilots maintain spatial orientation during flight. Furthermore, tactile signals have been used to help subjects perform breathing exercise to improve their effectivity [19].

The main aim of this study was to investigate whether tactile guided slow deep breathing effectively increases arterial oxygen saturation during acute hypobaric hypoxia. For this purpose, we compared the effect of tactile guided slow deep breathing and spontaneous breathing on arterial oxygen saturation. We measured the changes in arterial oxygen saturation indirectly through pulse oximetry (SpO_2_). To investigate whether tactile guided slow deep breathing is also effective during a cognitive task, we compared SpO_2_ at rest and during a cognitive task. Hypoxia was induced by exposing the participants to a simulated altitude of 4,572 m in a hypobaric chamber. In addition, participants also subjectively rated their alertness and hypoxia symptoms.

In this repeated measures study, we addressed the following research questions: 1) does tactile guided slow deep breathing under hypobaric hypoxia increase SpO_2_ compared with spontaneous breathing, especially when the participants are performing a cognitive task?, 2) how does tactile guided slow deep breathing affect the participants’ alertness and hypoxia symptoms?, and 3) how usable do participants find tactile breathing guidance?

We expected SpO_2_ to be higher during tactile guided slow deep breathing than during spontaneous breathing even while participants were performing a cognitive task. We also expected that the increase in SpO_2_ during tactile guided slow deep breathing would increase alertness and reduce the number of hypoxia symptoms.

## Material and methods

### Participants

Twelve male military pilots (mean age ± SD, 37 ± 9.1 years and mean total flight hours 1,337 ± 1,114) of the Royal Netherlands Air Force volunteered for the experiment. The pilots were recruited by an invitation email explaining the study, which was sent to all pilots who had participated in previous hypoxia studies and had given their consent to be contacted for participation in further studies, and to pilots who had scheduled their yearly medical examination at the Centre for Man in Aviation during the study period. A reminder email was sent two weeks after the initial email. All pilots were recruited between November 2022 and January 2023. To be eligible for inclusion, pilots needed to have passed their mandatory yearly medical examination and be declared “fit to fly”. Pilots were excluded if they had been consecutively flying at altitudes higher than 2,438 m for longer than a week in the three-month period before the study started. All participating pilots had previous experience in the hypobaric chamber and were familiar with hypoxia symptoms. On the test day, the researcher explained the experimental procedure to the participants and answered any questions about the study. The participants then voluntarily signed the informed consent form. The study protocol was approved in advance by the Medical Ethical Committee of the Amsterdam Academic Medical Centre (2022_0560).

To determine the a priori sample size, we made a calculation using the software G*Power (Version 3.0; Berlin, Germany). The sample size was calculated using reported data by Bilo et al. [9] who investigated the ventilatory and hemodynamic effects of slow deep breathing in normal subjects at high altitude. The mean ± SD SpO_2_ of spontaneous breathing (80.2 ± 7.7) and slow deep breathing (89.5 ± 8.2) under hypoxic conditions were used in a repeated-measures within-factors statistical analysis. A sample size of 12 participants was needed to achieve a power of 80% from four SpO_2_ measurements (two SpO_2_ measurements for each breathing condition) with a correlation of 0.5.

### Intervention

Hypoxia was induced in a hypobaric chamber at a simulated altitude of 4,572 m. The study had two independent variables: breathing (spontaneous versus tactile guided slow deep breathing) and task (rest versus cognitive task). This resulted in four conditions:

1. Spontaneous breathing at rest (Spont-Rest)
2. Spontaneous breathing while performing a cognitive task (Spont-Task)
3. Tactile guided slow deep breathing at rest (Guided-Rest)
4. Tactile guided slow deep breathing while performing a cognitive task (Guided-Task)

Slow deep breathing was performed at a rate of six breaths per minute, which has been shown to improve blood oxygenation^3^. It was guided using tactile signal that was generated by vibration motors that were integrated into a base layer shirt. In order to allow for deep inhalation during this phase the participants received instructions regarding diaphragmatic breathing. The duration of both inhalation and exhalation was 4.5 seconds. There was a 0.5-second pause between each breathing phase. An increase in the vibration frequency (from 0 rpm to 12,000 rpm) told participants that they needed to inhale, and a decrease in the vibration frequency (from 12,000 rpm to 0 rpm) told the participants that they needed to exhale (see the ‘equipment’ section for details on the vibration system). We chose this vibration pattern based on the preference of seven out of ten people who participated in a short pilot study comparing different vibration signals and patterns.

The duration of each condition was ten minutes and spontaneous breathing was performed first and served as a baseline. We chose intervals of ten minutes for each measurement for two reasons: 1) because this was sufficient to observe the change and for the physiological parameters to reach a steady state and 2) because it allowed us to collect sufficient number of breaths for the analysis, especially for the tactile guided slow deep breathing condition where the number of breaths per minute was six.

### Study variables

For the first study question, blood oxygen saturation (SpO_2_) was the primary dependent variable. Additional physiological parameters were heart rate (HR), respiratory frequency (RF), minute ventilation (V_E_), tidal volume (V_T_), end-tidal oxygen partial pressure (PetO_2_), and end-tidal carbon dioxide partial pressure (PetCO_2_).

For the second study question, the participants rated their level of alertness using the Stanford Sleepiness Scale (SSS) [20]. The SSS is a seven-point Likert-type scale that ranges from “feeling active, vital, alert, or wide awake” (score = 1) to “no longer fighting sleep, sleep onset soon, and having dream-like thoughts” (score = 7). Hypoxia symptoms were marked by the participants from a list of general hypoxia symptoms as previously reported [21–23]. These symptoms were tiredness, cold/hot flashes, tingling of the fingers, shortness of breath, fast breathing, dizziness, nausea, headache, loss of concentration, reduced visual acuity, and decreased color vision. The participants could also add any symptoms that were not mentioned on the list.

For the third study question, usability of tactile breathing guidance was assessed using a questionnaire of ten questions. Seven questions were answered using a visual analogue scale (VAS) (Table 1). The VAS consisted of a 100-mm horizontal line with opposite claims at each end. Study participants were asked to make a mark on the line that represented their opinion, and the VAS was scored by measuring how many millimeters the mark was from the left end of the line. In addition, the participants indicated in a “yes” or “no” answer whether they could follow the vibration pattern all the time. If they answered with a “no” they needed to indicate in which condition they could not follow the vibration pattern (rest period or cognitive task). The participants also needed to indicate whether the vibration pattern distracted them.

**Table 1:** Usability questionnaire. Overview of the seven questions and the end-point definitions of the VAS line.

Question	Left end point	Right end point
Could you feel the vibration signal?	Could not feel at all	Could feel very well
Was the vibration signal intensity strong enough?	Not strong at all	Strong enough
Was the vibration sensation annoying?	Very annoying	Not annoying at all
Was the vibration signal intuitive?	Not intuitive at all	Very intuitive
Was it easy to adjust your breathing to the vibration pattern?	Not easy at all	Very easy
Was it easy to follow the vibration pattern again?	Not easy at all	Very easy
How large was the effect of the vibration pattern on the execution of the cognitive task?	No effect at all	A large effect

### Equipment

The hypobaric chamber used in this study was at the Royal Netherlands Air Force Center for Man in Aviation located in Soesterberg, The Netherlands. This cylindrical chamber is 12.5 m long and 3.0 m wide. During ascent, a vacuum pump sucks air out of the chamber, lowering the pressure until it simulates that of the desired altitude.

**Fig 1:**
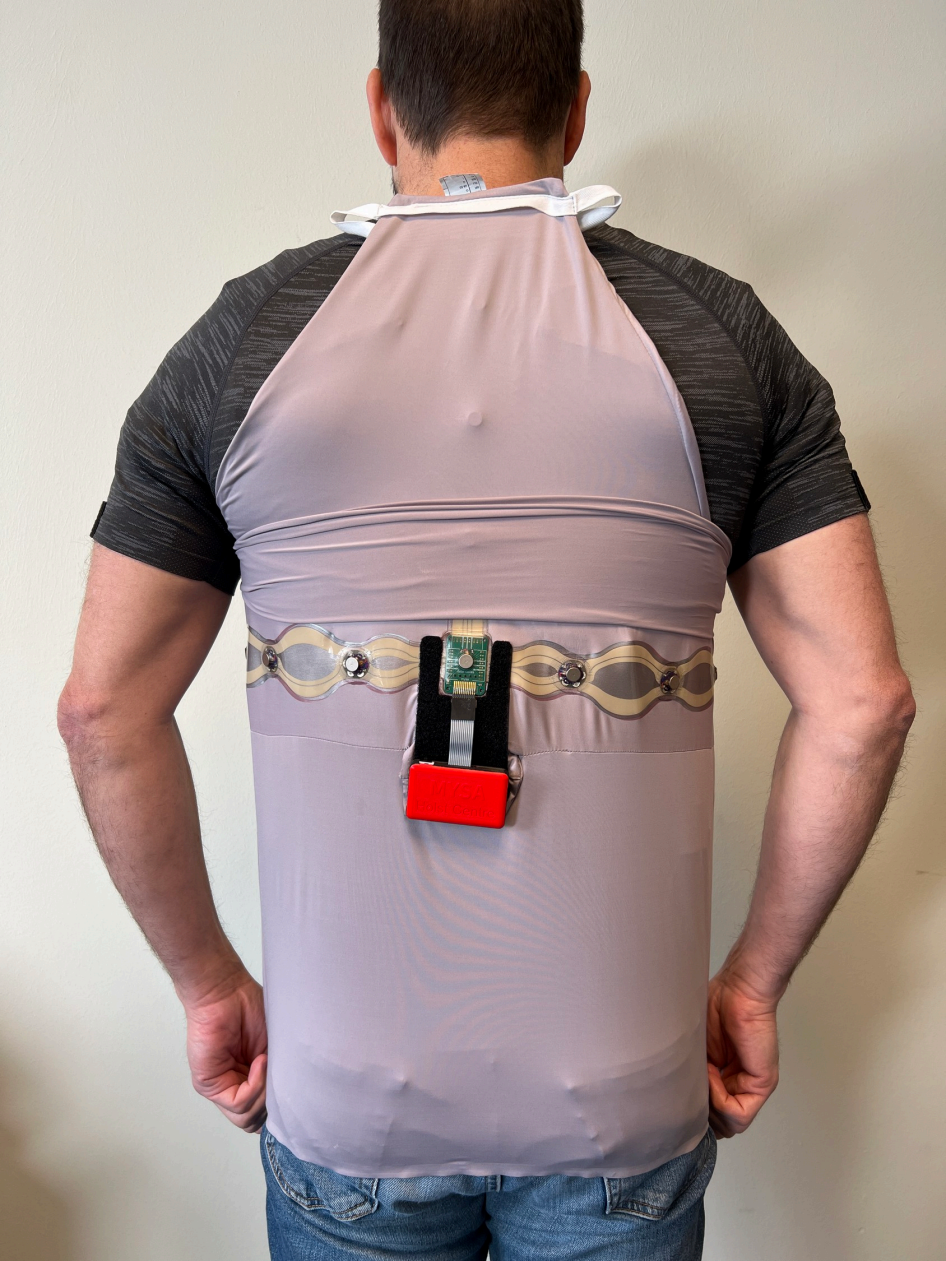
The MYSA system. Location of the six vibration motors across the middle of the back and the control box (red) containing the printed circuit board and battery.

Breathing was guided using the MYSA system (Touchwaves B.V, Eindhoven, The Netherlands). The system consisted of a printed circuit board (Arduino Feather 3244, Arduino, Somerville, MA, US), vibration motors (LilyPad Vibe Board, DEV-11008S, parkFun Electronics , Niwot, USA), and a control app (Bluefruit Connect, Adafruit Industries, New York, USA). Six vibration motors were seamlessly integrated into a shirt across the middle of the back (Fig 1). The vibration motors were 10 mm wide and 3.4 mm high and weighed 1.2 g. The voltage range of the motors was 2.5–3.8 V. The motors had a maximum of 12,000 rpm and a vibration amplitude of 0.8 Gs.

An Oxycon mobile breath-by-breath apparatus (CareFusion GmbH, Hoechberg, Germany) was used to measure and record the physiological parameters. During testing, participants wore an oronasal mask (7400 Series, Hans Rudolph Inc., Shawnee, KS, USA). The dead-space was 75 ml in the medium mask and 80 ml in the large mask. SpO_2_ and HR were measured using a Nonin 8000R forehead reflectance sensor (Nonin Medical, Inc., Plymouth, MN) that was connected to the Oxycon mobile. The physiological data were displayed, stored, and processed using the JLab 5.72 software (Becton, Dickinson and Company, Franklin Lakes, USA).

**Fig 2:**
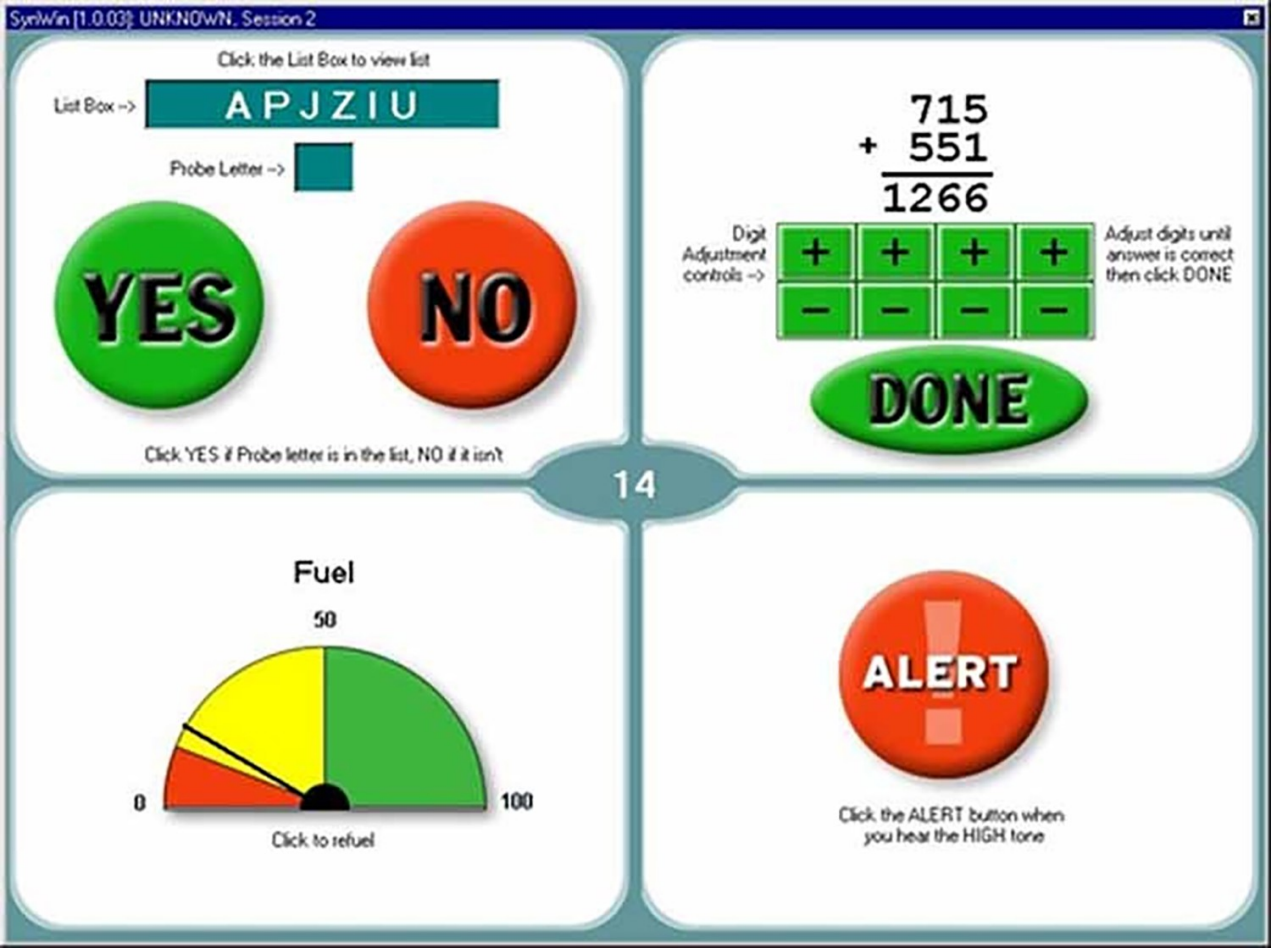
SynWin task battery.

The SynWin [24] cognitive task battery (Fig 2) (version 1.2.24, Activity Research Services, Chula Vista, USA) was used as the cognitive task. The purpose of performing the SynWin was to keep the participants occupied during both breathing conditions, in order to determine if the tactile breathing guidance was also effective while a pilot is occupied with a cognitive task. The SynWin is a computerized test used to assess cognitive processes such as working memory, visual perception, and multitasking. It consists of four continuous tasks, which are presented on the screen in separate quadrants: a memory task in the upper left quadrant, an arithmetic task in the upper right quadrant, a visual monitoring task in the lower left quadrant, and an auditory monitoring task in the lower right quadrant. In the middle of the screen, the composite score of all four tasks is displayed. In the memory task, the participants had to memorize four letters. The duration of the SynWin test was 10 minutes.

### Procedure

On test day, before starting the test session, the participants received instructions and practiced the SynWin test and the slow deep breathing. The SynWin test was practiced until the number of mistakes made in the memory and arithmetic tasks did not exceed 10% of the total answers. The tactile guided slow deep breathing was practiced until the participants could follow the vibration patterns easily. The participants were told that, during the test sessions, the SynWin test was their primary objective, and that their goal was to reach their highest score from the practice sessions. Before starting the ascent, the oxygen sensor of the Oxycon mobile was calibrated.

**Fig 3:**
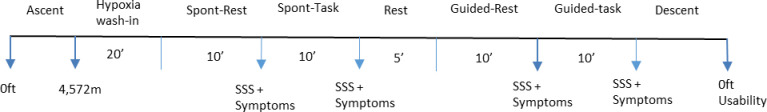
The experimental design. Spont-Rest = spontaneous breathing at rest; Spont-Task = spontaneous breathing while performing a cognitive task; Guided-Rest = tactile guided slow deep breathing at rest; Guided-Task = tactile guided slow deep breathing while performing a cognitive task. The durations of each period in minutes are indicated under the timeline. SSS = Stanford Sleepiness Scale, Symptoms = hypoxia symptoms, Usability = Usability questionnaire.

The experimental design and timing of measurements are reported in Fig 3. Ascent to altitude was at a rate of 914 m per minute. Once 4,572 m was reached, a 20-minute hypoxia “wash-in” period was started to induce an initial physiological reaction to hypoxia and to reach a steady state of breathing and SpO_2-_[25]. During this period, the environmental conditions in the hypobaric chamber (temperature, humidity, and barometric pressure) were fed into the Oxycon mobile, the flow sensor of the Oxycon mobile was calibrated, the participant was fitted with the Oxycon mobile, and the physiological data were measured. There was a five-minute break between the spontaneous breathing and the tactile guided slow deep breathing conditions.

Before starting and after completing the Spont-Task and Guided-Task conditions, the participants rated their state of alertness using the SSS and reported the hypoxia symptoms that they experienced at that moment. After completing the last condition (Guided-Task), the pressure in the hypobaric chamber was increased (700 m/min) to ambient pressure. Afterwards, the participants completed the questionnaire on the usability of the tactile breathing guidance.

### Data analysis

Data were analyzed using IBM SPSS 28. For the analysis, the average of all physiological parameters was calculated from the beginning till the end of each condition. The normality of the data was checked using the Kolmogorov–Smirnov test, and non-normally distributed data were analyzed with a non-parametric Friedman test. A two-way (breathing × task) repeated measures analysis of variance was used to test whether the physiological parameters (SpO_2_, HR, RF, V_E_, V_T_, PetO_2,_ and PetCO_2_) depended on breathing (spontaneous versus tactile guided slow deep breathing) and task (rest versus cognitive task). Differences in SSS ratings between the conditions were analyzed using the Friedman test. Frequency counts per participant were drawn from the lists of reported hypoxia symptoms and the differences in the number of reported hypoxia symptoms between the conditions were compared using a Friedman test. Descriptive statistics were used to analyze the results of the usability questionnaire. The level of significance for all comparisons was set at *p* < .05 and all results are presented as mean ± SD unless noted differently.

## Results

### Changes in blood oxygen saturation (SpO_2_)

The SpO_2_ was significantly higher (*F*(1, 11) = 24.269; *p* <.001; η_p_^2^ = .688) during tactile guided slow deep breathing than during spontaneous breathing (Table 2). No significant main effect was found on SpO_2_ for task (*F*(1, 11) = 3.703; *p* = .081; η_p_^2^ = .252) and no interaction was found between breathing and task (*F*(1, 11) = 2.754; *p* = .125; η_p_^2^ = .200).

**Table 2:** Physiological data. Mean and standard deviation of SpO_2_ and the physiological parameters during the four conditions.

	Spontaneous breathing		Slow deep breathing	
Parameter	Rest	Cognitive Task	Rest	Cognitive task
SpO_2_ (%)	78 ± 4.2	78 ± 3.0	88 ± 5.3^a^	85 ± 5.9^a^
HR (1/min)	75 ± 10.8	77 ± 16.3	70 ± 10.0^a^	73 ±12.6^a^
RF (1/min)	12 ± 2.6	16 ± 3.6	7 ± 1.1^a^	10 ± 2.3^a^
V_E_ (L/min)	10 ± 1.9	11 ± 1.9	12 ± 3.7	10 ± 2.0
V_T_ (L)	1.0 ± 0.2^b^	0.8 ± 0.2	1.8 ± 0.5^a^^,^ ^b^	1.2 ± 0.4^a^
PetO_2_ (kPa)	6.2 ± 0.4	6.4 ± 0.4	6.8 ± 0.7^a^	6.6 ± 0.5^a^
PetCo_2_ (kPa)	3.9 ± 0.3	3.8 ± 0.2	3.6 ± 0.4^a^	3.6 ± 0.4^a^

SpO_2_: oxygen saturation, HR: heart rate, RF: respiratory frequency, V_E_: minute ventilation, V_T_: tidal volume, PetO_2_: end tidal O_2_, PetCO_2_: end tidal CO_2_.

a. Significant at *p* < .05 level between spontaneous and tactile guided slow deep breathing conditions

b. Significant at *p* < .05 level between rest and cognitive task conditions

Measurement of the respiratory parameters (Table 2) showed that the HR was significantly lower (*F*(1, 11) = 18.773; *p* = .001; η_p_^2^ = .631) during tactile guided slow deep breathing than it was during spontaneous breathing. No main effect was found for task on HR (*F*(1, 11) = 3.549; *p* = .086; η_p_^2^ = .244) and no interaction was found between breathing and task (*F*(1, 11) = .039; *p* = .846; η_p_^2^ = .004). RF was significantly lower (*F*(1, 11) = 58.232; *p* <.001; η_p_^2^ = .841) during tactile guided slow deep breathing than during spontaneous breathing and was significantly higher (*F*(1, 11) = 20.986; *p* <.001; η_p_^2^ = .656) during the cognitive task than during rest. For RF, there was no significant interaction between breathing and task (*F*(1, 11) = .403; *p* = .539; η_p_^2^ = .035). For V_E_, there was no significant main effect of breathing or task (*F*(1, 11) = .502; *p* =.493; η_p_^2^ = .044 and *F*(1, 11) = 1.169; *p* = .303; η_p_^2^ = .096, respectively) and no significant interaction between breathing and task (*F*(1, 11) = 4.199; *p* = .065; η_p_^2^ = .276). There was a main effect of breathing and task on V_T_ (*F*(1, 11) = 18.260; *p* = .001; η_p_^2^ = .624 and *F*(1, 11) = 31.201; *p* < .001; η_p_^2^ = .739, respectively). For V_T_, an interaction was also found with breathing and task (*F*(1, 11) = 5.550; *p* = .038; η_p_^2^ = .335). A post-hoc analysis comparing simple main effects with Bonferroni confidence interval adjustment revealed that the V_T_ was significantly larger during the rest condition than during the cognitive task condition in both the spontaneous breathing and tactile guided slow deep breathing conditions (*p* = .005 and *p* < .001, respectively).

A main effect was found for breathing on PetO_2_ and PetCO_2_ (*F*(1, 11) = 5.060; *p* = .046; η_p_^2^ = .315 and *F*(1, 11) = 6.255; *p* = .029; η_p_^2^ = .326, respectively), with PetO_2_ increasing during tactile guided slow deep breathing compared with during spontaneous breathing and PetCO_2_ decreasing during tactile guided slow deep breathing compared with during spontaneous breathing. No significant main effect was found for task on PetO_2_ and PetCO_2_ (*F*(1, 11) = .032; *p* = .862; η_p_^2^ = .003 and *F*(1, 11) = .812; *p* = .387; η_p_^2^ = .069, respectively) and no interaction was found between breathing and task (*F*(1, 11) = 3.498; *p* < .088; η_p_^2^ = .241 and F(1, 11) = .337; p = .574; ηp2 = .030).

**Fig 4:**
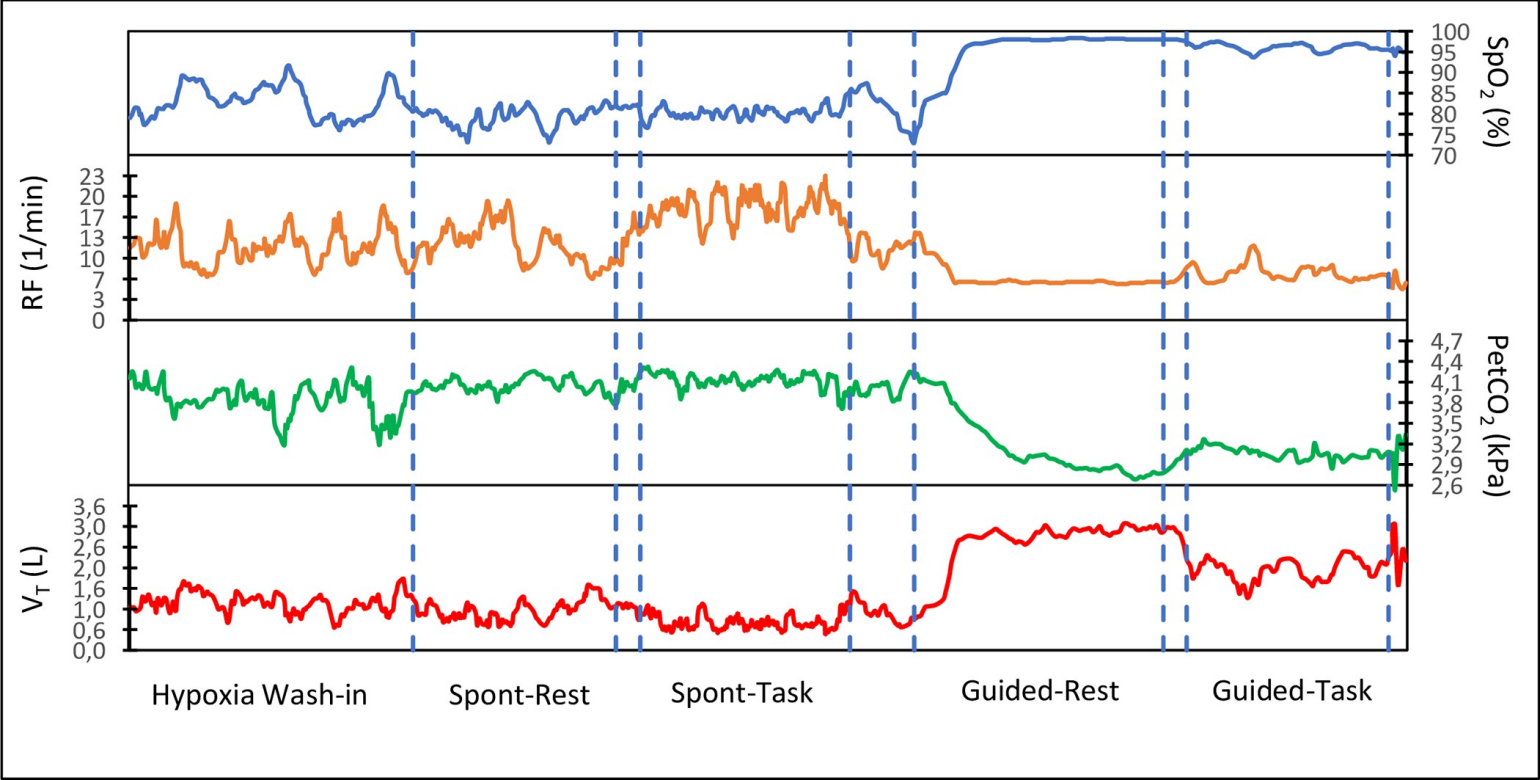
Time series including physiological data and measurement durations. The respiratory frequency (RF) of one participant during the different breathing conditions and the corresponding effect on oxygen saturation (SpO_2_), tidal volume (V_T_) and end-tidal carbon dioxide partial pressure (PetCO_2_). Each condition is indicated at the bottom and the dotted vertical lines indicate the duration of the four conditions. Spont-Rest = spontaneous breathing at rest, Spont-Task = spontaneous breathing while performing a cognitive task, Guided-Rest = tactile guided slow deep breathing at rest, Guided-Task = tactile guided slow deep breathing while performing a cognitive task.

A time series for the RF, SpO_2_, V_T_, and PetCO_2_ is shown for one participant during the different conditions in Fig 4. Notice that the SpO_2_ started to rise and PetCO_2_ decreased the moment the participant started slow deep breathing, and both remained at the approximately the same level as long as the RF and V_T_ were maintained. The data of the RF plot show that maintaining six breaths per minute was more challenging during the cognitive task than during rest and that the increased RF lowered the V_T_, which lowered the SpO_2_ and increased PetCO_2_ compared with the rest condition.

### Effect on alertness and hypoxia symptoms

The effect of tactile guided slow deep breathing on alertness and hypoxia symptoms are presented in Table 3. The non-parametric Friedman test analysis showed no significant differences in SSS ratings (*χ*^*2*^(3) = 1.418, *p* = .701) at the start and at the end of the Spont-Task condition compared with those at the start and end of the Guided-Task condition. In addition, there was no significant effect of tactile guided slow deep breathing (χ^2^(3) = .495, p = .920) on the number of hypoxia symptoms reported at the start and at the end of the Spont-Task condition compared with at the start and at the end of the Guided-Task condition.

**Table 3:** Stanford Sleepiness Scale data. Median and interquartile range of Stanford Sleepiness Scale ratings and number of hypoxia symptoms at the start and at the end of spontaneous breathing while performing a cognitive task (Spont-Task) and tactile guided slow deep breathing while performing a cognitive task (Guided-Task).

	Spont-Task				Guided-Task			
	Start		End		Start		End	
Parameter	Median	IQR	Median	IQR	Median	IQR	Median	IQR
Stanford Sleepinees Scale	3	2–3	3	2–3	3	3–3	3	2–4
Number of hypoxia symptoms	3	2–4	4	2–5	3	3–4	4	2–5

### Usability of tactile breathing guidance

**Fig 5:**
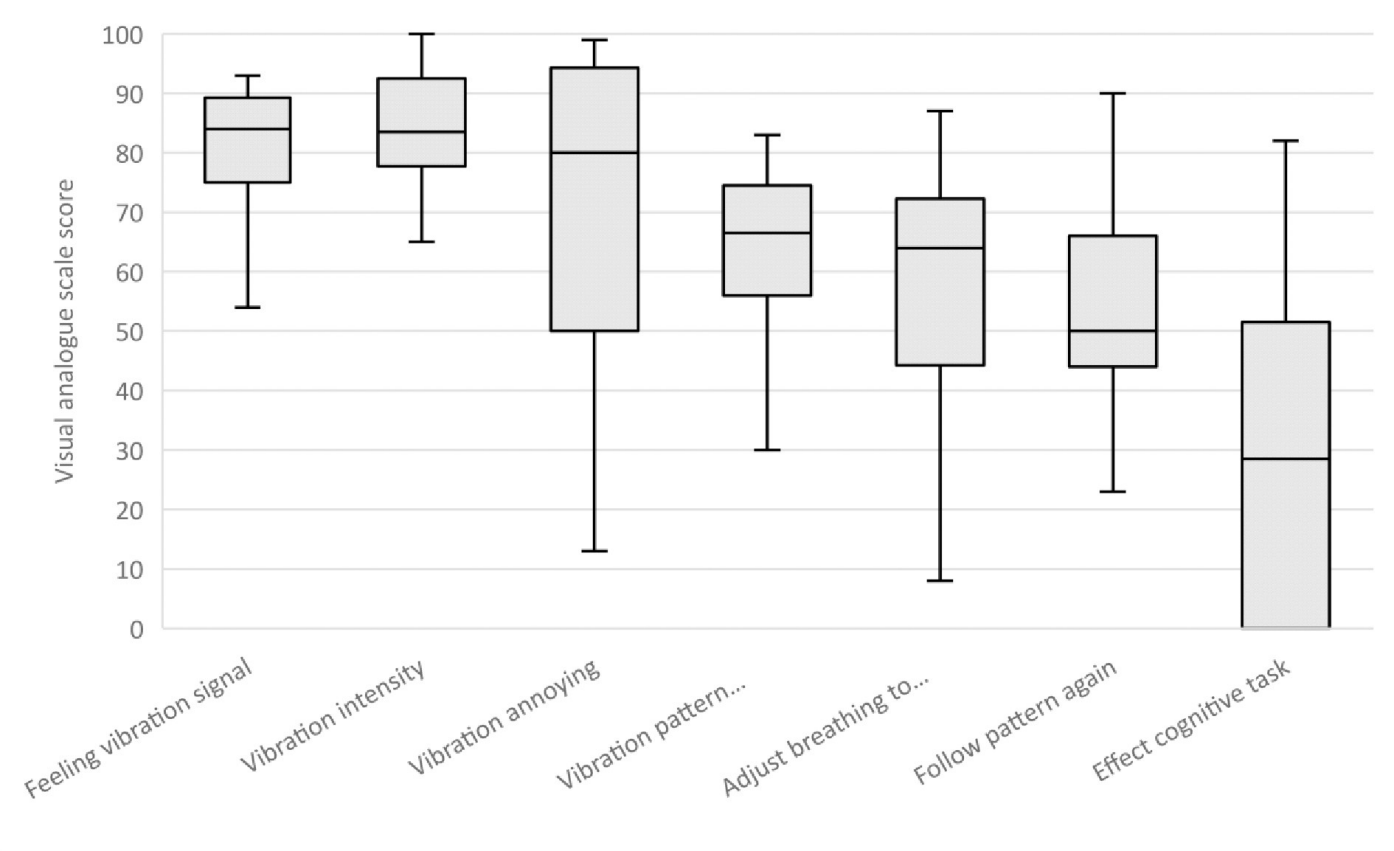
Questionnaire results. The median, inter-rate quartile, minimum score, and maximum score of the visual analogue scale for each of the usability questions. A score of 0 corresponded to the claim on the left of the visual analogue scale and a score of 100 corresponded to the claim on the right of the visual analogue scale.

The VAS ratings in Fig 5 show that participants gave median VAS scores of 80 or higher for the questions on whether they were able to feel the vibration signal, the strength of the signal, and if the vibration signal was annoying. They gave lower scores (median between 60 and 70) for the intuitivity of the vibration pattern and their ability to adjust their breathing to the vibration pattern. On average, the participants did not find it very easy to start following the vibration pattern again (median score of 50).

In the “yes” or “no” questions, 11 participants (92%) indicated that they could not follow the vibration pattern all the time. Ten of these participants (91%) reported that they could not follow the vibration pattern while performing the cognitive task; the remaining participant (9%) reported that he could not follow the vibration pattern either during the rest condition or during the cognitive task condition. Eight participants (67%) reported that they were distracted by the vibration pattern. However, five of those participants (63%) reported that the vibration pattern did not have a large effect on their performance of the cognitive task (VAS score < 50).

## Discussion

Our results demonstrate that tactile guidance of slow deep breathing significantly increased SpO_2_ during hypobaric hypoxia compared with spontaneous breathing, independent of whether the participants were at rest or engaged in a cognitive task. We found no differences in alertness (measured by SSS ratings) and in the number of reported hypoxia symptoms between tactile guided slow deep breathing and spontaneous breathing. Regarding the usability of tactile breathing guidance, the participants were positive about the intensity and sensation of the vibration signal, but most found it difficult to follow the vibration pattern during the cognitive task.

The main effect we observed of tactile guided slow deep breathing on SpO_2_ is in line with the findings of Bilo et al. [9], who used high and low sound tones to guide slow deep breathing. The increase in SpO_2_ we observed was probably due to the improved ventilation efficiency as indicated by the increased PetO_2_ and decreased PetCO_2_. The 80% increase in V_T_ during the Guided-Rest condition compared with during the Spont-Rest condition and the 50% increase during the Guided-Task condition compared with during the Spont-Task condition may have improved ventilation efficiency by reducing anatomical dead space in the lungs, optimizing alveolar ventilation, and increasing blood flow to areas of the lungs that are normally less well perfused [9, 10].

Manifestation of hypoxia symptoms is associated with the decrease in SpO_2_ level [23, 26]. Previous studies [8, 9] have suggested that the SpO_2_ increase after slow deep breathing would effectively cope with the effects of altitude-induced hypoxia. However, our results did not support this assumption, as the effects of hypoxia on alertness and hypoxia symptoms did not diminish with the increase in SpO_2_. This may be due to the significant decrease in PetCO_2_ observed during tactile guided slow deep breathing compared with during spontaneous breathing. A decrease in PetCO_2_ can cause similar symptoms to those caused by a decrease in SpO_2_ [3, 27]. This could probably be the result of the increase in cerebral vasoconstriction caused by the decrease in PetCO_2_ [27], negatively affecting cerebral blood flow [28] and cerebral oxygenation [29]. A recent hypoxia study [30] reported that a hyperventilation-induced decrease in PetCO_2_ led to more hypoxia symptoms being reported than spontaneous breathing did, even though SpO_2_ was significantly higher during hyperventilation. These findings, together with those of our study, may emphasize the importance of PetCO_2_ in the development of hypoxia symptoms.

The usability questionnaire revealed that the participants could feel the vibration signal and perceive it even under hypoxia with decreased alertness and during the cognitive task. The participants did not find the vibration motors across their back annoying, which is in line with the results of Haans et al. [31], who reported that vibration motors were less annoying on the back than on other parts of the body. Short pre-training with the MYSA system and with slow deep breathing might explain why the participants did not find the vibration signal very intuitive and were less able to adjust their breathing to the vibration pattern. It may also explain why the participants were distracted by the vibration pattern when performing the cognitive task.

This is a preliminary pilot study assessing whether tactile guided slow deep breathing can increase SpO_2_ during hypoxia. We measured the effect of tactile guided slow deep breathing on SpO_2_, alertness, and hypoxia symptoms, and evaluated its usability under controlled conditions. Future studies may examine the effect of tactile guided slow deep breathing on these variables under dynamic conditions that represent real operational flight; for example, in a flight simulator, exposing aircrew members to multiple external stressors such as vibration, noise, and heat and having participants wear flight gear such as survival vests and ballistic plates. We measured the number of hypoxia symptoms but not the severity of the symptoms. It is therefore possible that the number of reported symptoms remained the same but that the severity of the symptoms changed, indicating an effect of the intervention which could not be determined by measuring the number of symptoms alone. Future studies should also measure the severity of symptoms.

We have several recommendations based on our findings. First, our participants had difficulty maintaining the six breaths per minute pattern during the cognitive task. Bernardi et al. [32] have shown that one month of deep and slow breathing training was sufficient to improve breathing performance. Therefore, we recommend training with the breathing technique to improve tactile breathing guidance during cognitive tasks. Second, the Royal Netherlands Air Force aircrew members are taught during their hypoxia training that the severity of their hypoxia symptoms are dependent on the level of SpO_2_, but this study and previous studies [14, 30] have shown that PetCO_2_ levels play a central role in the development of hypoxia symptoms even when SpO_2_ is elevated. Therefore, we recommend that, during their hypoxia training, aircrew members be made aware that not only SpO_2_ but also PetCO_2_ can affect hypoxia symptoms. Third, there is growing interest in wearable technology for in-flight monitoring of aircrew, which provides physiological data that may help predict pilots’ status and promote flight safety and efficiency [27, 33, 34]. Future studies can look into the integration of physiological monitoring instruments with tactile guided slow deep breathing to provide a detection, warning, and guidance system for aircrew.

## Conclusion

Our results demonstrate that tactile guided slow deep breathing increased SpO_2_ significantly compared with spontaneous breathing under hypoxia, both at rest and during a cognitive task. The increase in SpO_2_ after tactile guided slow deep breathing did not alter alertness and hypoxia symptoms, emphasizing the role PetCO_2_ plays in the development of hypoxia symptoms. Attitudes towards the intensity and sensation of the vibration signal were positive, but participants had difficulty following the vibration pattern during the cognitive task.

## Supporting information

S1 Data (7Z)
